# Monteggia fractures: analysis of patient-reported outcome measurements in correlation with ulnar fracture localization

**DOI:** 10.1186/s13018-022-03195-1

**Published:** 2022-06-07

**Authors:** Eric Tille, L. Seidel, A. Schlüßler, Franziska Beyer, P. Kasten, O. Bota, A. Biewener, J. Nowotny

**Affiliations:** 1 University Centre for Orthopaedic, Trauma- and Plastic Surgery (OUPC), University Hospital Carl Gustav Carus, Technical University Dresden, Fetscherstraße 74, 01307 Dresden, Germany; 2 Centre for Translational Bone, Joint and Soft Tissue Research, Technical University Dresden, Dresden, Germany; 3 Orthopaedic Surgery Centre (OCC), Tübingen, Germany

**Keywords:** Monteggia fracture, Monteggia-like lesion, Elbow fracture, Patient-reported outcome, Fracture treatment

## Abstract

**Background:**

Monteggia fractures and Monteggia-like lesions result after severe trauma and have high complication rates. Preliminary biomechanical studies suggested a correlation between ulnar fracture localization and clinical result.

**Objectives:**

Key objective was to evaluate whether the site of the ulnar fracture can be correlated to clinical outcome after open reduction and internal stabilization.

**Methods:**

In a retrospective, monocentric study 35 patients who underwent surgical treatment after suffering a Monteggia injury or Monteggia-like lesion were included. Fractures were classified according to Bado and Jupiter, the site of the fracture location at the proximal ulna and regarding the potential accompanying ligamentary injury. In a follow-up examination validated patient-reported outcome measures and functional parameters were evaluated. Furthermore, treatment strategy and complications were analysed.

**Results:**

Mean patient age was 51.9 years (± 18.0). 69% were females (*n* = 24). Follow-up took place after 50.5 months (± 22.1). Fractures were classified according to Bado (I:2, II:27, III:4, IV:2). Bado II-fractures were further classified according to Jupiter (A:7, B:16, C:3, D:1). Cases were divided into subgroups depending upon the distance of the ulnar fracture site in respect to its distal endpoint (A: < 7 cm and B: > 7 cm). Average overall MEPS was 84.1 (± 19.0). Oxford elbow score and DASH were 37.2 (± 10.5) and 20.4 (± 20.5). Average extension capability reached − 7° (± 7.5). Mean flexion was 134.8° (± 19.7). Average pain according to visual analogue scale was 1.6 (± 1.9). We found no differences between the subgroups regarding the PROMs. Subgroup A displayed a worse extension capability (*p* = 0.027) and patients were significantly older (*p* < 0.01). Comparing patients with and without fracture of the radial head, we observed no differences. Patients with an accompanying injury of the coronoid process displayed higher pain levels (*p* = 0.011), a worse functionality (*p* = 0.027) and overall lower scoring in PROM.

**Conclusion:**

The presented results suggest that in Monteggia fractures and Monteggia-like lesions, the localization of the ulna fracture can give a hint for its postoperative outcome. However, we could not confirm the hypothesis of an increasing instability in ulnar fractures located further distally (high severity of the potential ligamentous injury). Intraarticular fractures or injuries with a close relation to the joint have a worse prognosis, especially if the coronoid process is injured.

*Trial registration* Registration was done with ClinicalTrials.gov under NCT05325268.

## Introduction

A Monteggia fracture (MF) is defined as a fracture of the proximal ulna combined with a dislocation of the radial head. If additional fractures (i.e. of the radial head or the coronoid process) are present, injuries are being considered as Monteggia-like lesions (MLL). Grading of Monteggia injuries follows the Bado classification, which focuses on the direction of the radial head dislocation, whereby the ulna fracture can occur at very distinct locations [1]. The site of ulnar fracture is not being considered in present classifications.

In case of radial head dislocation, the joint capsule and further ligamentary structures between ulna and radius are likely to be injured. A hypothesis postulates that the ligament structures tear up to or even beyond the level of the ulna fracture which can be comparable to injuries of the syndesmosis at the ankle joint. In a preliminary biomechanical study of the authors, the stability in the proximal radio-ulnar joint (PRUJ) was directly related to the affected ligamentary structures between ulna and radius [2]. Depending on the injured ligamentary structures an increasing dislocation (primarily in posterior and lateral direction) was observed after successive resection up to the interosseous membrane. The ligamentary structures between ulna and radius distal to the fracture line potentially remained intact. Based on this assumption, it seemed interesting whether these findings could be verified in the clinical setting. The question raised was therefore, whether the site of the ulna fracture and its distance from the PRUJ in MF and MLL can be correlated to the postoperative outcome and the potential ligamentary damage.

## Methods

### Study design and patients

A retrospective, monocentric study at a university hospital was conducted. Analysing the hospital’s medical records a total of 40 consecutive patients were identified. Out of these, 35 were successfully recruited for this study. All patients had suffered a MF or a MLL. Thirty-three patients underwent surgical treatment at the University Center between January 2012 and December 2018. Mean age was 51.9 years (SD: ± 18, min: 22, max: 84), with a mean follow-up time of 50.5 months (SD: ± 22.1). Twenty-four patients (69.0%) were females and eleven patients (31.0%) were males. In fifteen cases (43.0%) the right arm was injured. Twenty-one patients (62.9%) suffered from an additional fracture of the radial head and were therefore classified as MLL. In 13 cases (37.1%) we observed a fracture of the coronoid process. Demographic data are shown in Table 1.

**Table 1:** Demographic facts of participating patients. Values given as average ± standard deviation

Patient description			
Age (years)	51.9 ± 18.0	Min: 22	Max: 84
Follow-up (months)	50.5 ± 22.1	Min: 16	Max: 88
Gender	24 female (68.6%)	11 male (31.4%)	
Injured Limb	15 right (42.9%)	20 left (57.1%)	

Approval for the study was granted by the local ethics committee (EK 77032018).

### Surgical technique

All procedures were performed under regional anaesthesia (interscalene brachial plexus blockade) and/or in general total intravenous anaesthesia. Patients were treated in a supine position. A tourniquet was placed at the upper arm. Three different surgeons carried out the procedures, however, one surgeon did 22 surgeries. Open reduction and internal fixation of the ulna was performed using locking compression plates (LCP) with 4–10 holes. Accompanying fractures of the radial head were, if necessary, addressed by either osteosynthesis (screws, plating), partial resection or implantation of an endoprosthesis. Injuries of the coronoid process were treated whit a lag screw or a neutralizing plate in four cases.

### Outcome

All patients and radiological findings were examined by one independent researcher who was not involved in the surgical care. The evaluated patient-reported outcome measures included Mayo Elbow Performances Score (MEPS), Oxford Elbow Score (OES), Disabilities of Arm, Shoulder and Hand (DASH), pain on visual analogue scale (VAS) and postoperative satisfaction [3–5]. The values for the different scores used in this study are displayed in Table 2. Furthermore, functional parameters such as the range of motion (ROM) measured by means of a manual goniometer were assessed. Strength, posterolateral instability and valgus-/varusstress were clinically evaluated.

**Table 2:** Threshold of the different scores used in this clinical investigation

Score	Excellent	Good	Satisfactory/fair	Poor
MEPS	90–100	75–89	60–74	< 60
OES	40–50	30–39	20–29	< 20
DASH	0–5	6–15	16–35	> 35

Radiographic images were classified according to Bado and Jupiter [1, 6]. In addition, accompanying injuries (i.e. fractures of the radial head) and the fracture site of the ulna were categorized. The distance between the proximal tip of the Olecranon and the fracture line of the ulna was measured. Subsequently, patients were divided into two subgroups depending on fracture localization and tissue damage. Group A included all patients with a distance of less than 7 cm and therefore comprises injuries with close proximity or directly affecting the joint (i.e. fractures of the coronoid process). Group B included all patients with a greater distance. The 7 cm cut-off was chosen since it resembles the distal insertion of the oblique chord [2, 7, 8]. In addition, subgroups with injuries of the radial head or the coronoid process were evaluated.

### Statistical analysis

Statistical analysis was performed using SPSS Statistics software (version 27; IBM, Armonk, NY, USA). In addition to the descriptive statistics, the differences between the preoperative and postoperative mean values were evaluated using Kruskal–Wallis test for unpaired groups, Mann–Whitney test for independent variables and regression analysis (significance level *p* < 0.05). All data are presented as mean or average with standard deviation as stated.

## Results

### Patients and indication

Table 3 summarizes the fracture classifications of the patient collective. Average distance of the ulna fracture, measured from the Olecranon tip, was 5.36 cm (SD: ± 2.3 cm, min: 2.8 cm, max: 11.4 cm). Twenty-nine patients were allocated to subgroup A with a close relation or directly affecting the ulnohumeral joint. Within this group the average patient age was significantly higher compared to subgroup B (*p* < 0.01). No further demographic differences were observed.

**Table 3:** Fracture classification in accordance to Bado and Jupiter

Fracture classification	*N* (%)
Bado I	2 (6.0%)
Bado II	28 (77.0%)
Jupiter A	7 (25.9%)
Jupiter B	16 (59.3%)
Jupiter C	3 (11.1%)
Jupiter D	1 (3.7%)
Bado III	3 (11.0%)
Bado IV	2 (6.0%)

### Analysis of intervention and complications

Plating of the ulna was performed using various angular stable, interlocking implants. Depending on fracture morphology 4–10 hole plates were used. In 21 cases imaging revealed an accompanying fracture of the radial head. All cases were addressed by either osteosynthesis [screws (10), plating (7)], partial radial head resection [2] or implantation of an endoprosthesis [1]. In one case the fracture was treated conservatively. Additional fractures of the coronoid process were observed in 13 cases. These were treated via lag screw or a neutralizing plate in four patients. In all other cases fragments were too small for refixation.

One patient needed revision surgery due to a local wound infection, which healed under local therapy. One patient needed revision surgery due to an insufficient reduction. In 11 cases complete, in one case partial removal of the hardware was performed after healing of the fracture. One patient suffered from a persisting decreased strength in the elbow and the wrist compared to the opposing side.

### Analysis of functional outcome parameters

The extension capability reached an average of − 7° (SD: ± 7.6°, min: − 20°, max: 10°), the average flexion was 134.8° (SD: ± 20.1, min: 70°, max: 150°), the average pronation 84.4° (SD: ± 14.2, min: 20°, max: 90°) and the average supination 77.0° (SD: ± 15.3°, min: 20°, max: 90°) at the time of the follow-up. There was no statistic difference regarding flexion, supination and pronation between the two subgroups. Extension was significantly worse in patients of subgroup A (− 9.1° ± 4.8° vs. B 5.0° ± 8.6°, *p* =  < 0.01). Patients with accompanying injuries of the radial head displayed no worse outcome. Statistical analysis however showed that these patients were significantly older (52.6 years ± 18.0 vs 44.1 years ± 17.7, *p* = 0.048). Patients with fractures of the coronoid process displayed a worse extension (− 11.1° ± 3.3° vs − 5.0° ± 8.4°, *p* = 0.027). Table 4 gives an overview over the functional outcome parameters.

**Table 4:** Range of Motion in (°) at time of follow-up

Functional criteria	Total	Subgroup A	Subgroup B	Radial head	Coronoid process
Extension	− 7.0 ± 7.5	− 9.1 ± 4.8	5.0 ± 8.6	6.9 ± 4.8	− 11.1 ± 3.3
		*p* = 0.027*		*p* = 0.845	*p* = 0.027*
Flexion	134.8 ± 19.7	132.4 ± 20.5	147.5 ± 4.3	129.4 ± 23.6	129.4 ± 23.2
		*p* = 0.059		*p* = 0.092	*p* = 0.466
Pronation	84.4 ± 14.0	83.9 ± 15.0	87.5 ± 4.3	83.5 ± 17.3	77.8 ± 23.3
		*p* = 0.921		*p* = 0.794	*p* = 0.315
Supination	77.0 ± 15.0	75.9 ± 15.9	83.8 ± 4.15	74.7 ± 17.7	70 ± 20.6
		*p* = 0.372		*p* = 0.306	*p* = 0.071

Values given in ° as average with standard deviation

*Significant result *p* < 0.05

### Analysis of PROMs

Evaluating the PROMs we observed an average overall MEPS of 84.1 (SD: ± 19.3, min: 35, max: 100), whereby in 18 cases an excellent (51.4%), in 10 a good (28.6%), in 4 a satisfactory (11.4%) and in 3 poor result (8.6%) was achieved. The DASH was 20.4 (SD: ± 20.8, min: 0, max: 69) (13 excellent [37.1%], 6 good [17.1%], 5 satisfactory [14.3%], 11 poor [31.4%]), while Oxford elbow score was 37.2 (SD: ± 10.7, min: 13, max: 48) (18 excellent [51.4%], 8 good [22.9%], 6 satisfactory [17.1%], 3 poor [8.6%]). There were no statistically significant differences between the analysed subgroups regarding the PROMs, VAS or patients´ satisfaction.

The evaluation of the fracture subclassification (Jupiter) revealed worse functional outcomes for patients who suffered a Jupiter A injury (extension: Jupiter A − 12.0° ± 4.0° vs Jupiter C − 2.0° ± 10.0° (*p* = 0.011); flexion: Jupiter A 115.0° ± 22.0° vs Jupiter B 138.0° ± 20.0° (*p* = 0.015), Jupiter A 115.0° ± 22.0° vs Jupiter C 143.0° ± 6.0° (*p* = 0.044)). In addition, long-term effects were observed. Patient’s satisfaction (+ 0.35 per month, *p* = 0.046) and OES-score (+ 0.19 per month, *p* = 0.015) rose continuously depending on time that had passed since the surgical treatment.

Patients with injuries of the radial head showed no worse outcome. However, patients with an injury of the coronoid process showed significantly higher pain levels (2.4 ± 1.7 vs 1.1. ± 1.9, *p* = 0.011) and lower overall satisfaction (62.3 ± 21.7 vs 86.4 ± 16.2, *p* < 0.01). Furthermore, all PROMs were evaluated significantly worse within these patients. An overview of the clinical outcome parameters is given in Table 5.

**Table 5:** Average results of evaluated PROMs

Score	Total	Subgroup A	Subgroup B	Fractured radial head	Fractured coronoid process
Mayo Elbow Performance Score (MEPS)	84.1 ± 19.0	82.4 ± 20.2	92.5 ± 7.5	81.7 ± 22.2	73.1 ± 23.1
		*p* = 0.312		*p* = 0.845	*p* = 0.015*
Disabilities of Arm, Shoulder and Hand (DASH)	20.4 ± 20.5	22.5 ± 21.5	10.2 ± 9.9	21.4 ± 22.8	32.4 ± 23.3
		*p* = 0.379		*p* = 0.879	*p* = 0.022*
Oxford Elbow Score (OES)	37.2 ± 10.5	36.0 ± 11.0	42.7 ± 5.2	36.5 ± 11.5	30.2 ± 12.0
		*p* = 0.312		*p* = 0.826	*p* = 0.014*
Subjective Satisfaction	77.4 ± 21.3	75.2 ± 22.2	88.3 ± 10.7	75.7 ± 23.2	62.3 ± 21.7
		*p* = 0.272		*p* = 0.607	*p* = 0.002*
Pain (VAS)	1.6 ± 1.9	1.7 ± 2.0	0.8 ± 0.9	1.9 ± 2.2	2.4 ± 1.7
		*p* = 0.454		*p* = 0.404	*p* = 0.011*

Values given as average with standard deviation

*Significant result *p* < 0.05

## Discussion

MF and MLL are rare but severe injuries of the elbow that benefit from adequate surgical treatment. If treated correctly, good to excellent mid- and long-term results are possible [9, 10]. However, even if early treatment is provided, the long-term outcome can be unsatisfying [11, 12].

MF have been classified in 1967 by Jose Bado [1]. As our study and multiple prior analysis have demonstrated, the predominant type—with a dorsal fracture and a dorsal dislocation of the radial head—is Bado II [12–14]. Only few other studies suggest Bado I to be the most common injury [15]. Adult Bado I fractures with intact radial head and absence of coronoid fracture usually result in satisfactory outcomes [14]. Jupiter established therefore a subclassification of the Bado II fracture type (A – D) [6]. In accordance with other studies most fractures observed were Jupiter IIB [6, 12–14, 16] injuries. Among the Bado II injuries, types IIA and IID show only moderate results compared to types IIB and IIC. Therefore, it is presumable, that poor function is mainly due to the presence of a fracture of the coronoid process [14, 17].

The reason for Bado II fractures being the predominant fracture type in adults is not entirely understood. As seen in a preliminary study, a possible reason might be an increasing instability of the elbow joint in posterior direction even after a presumably minor damage to the ligamentary structures [2]. According to the hypothesis that damage occurs from the joint capsule to the fracture line or beyond, a worse outcome for patients with distal fractures was expected. However, the presented results do not support this theory of an equivalent to a syndesmosis injury of the ankle. As seen in the aforementioned results, MF and MLL directly affecting or with a close relation to the joint do have a worse outcome. A main reason therefore might be the affection of the coronoid process which is crucial for joint stability. In the current study especially the extension capability, pain and clinical scores were significantly worse in these cases. This is congruent with previous findings in literature and thus supporting evidence of this knowledge [18–20]. Yet, the underlying reasons need to be further elucidated.

A potential cause might be that the forces of the trauma directly affecting the joint are being distributed onto a smaller area and therefore lead to more severe accompanying injuries such as fractures of the radial head or the coronoid process. In a farther proximal fracture the joint is potentially more severely damaged, such as the annular ligament which is crucial for stability. Also, further essential structures, such as the surrounding soft tissue or the cartilage, will suffer worse damage in proximal fractures. Distal fractures on the other hand could benefit from a larger area the impact is being distributed onto. Prior studies, however, have shown that the energy level of the trauma did not have any prognostic value [21]. Also in our study younger patients, who more often suffered high speed injuries/polytraumatic injuries displayed less severe MF and MLL. Older patients on the other hand, who often were injured after a direct fall onto the arm (considered to be a less severe trauma) suffered more often from severe MF and MLL, which might indicate the presence of an osteoporosis. Overall complex injury patterns have a negative long-term prognosis [12, 21]. It is therefore crucial to obtain best possible anatomic reduction to restore joint congruency. In our study MLL with an accompanying injury of the radial head displayed no worse functional outcome. Studies of Konrad et al. however have found an injury of the radial head to be a negative prognostic factor [12]. Moreover, it has been reported that fractures of the coronoid process—if possible—need to be addressed surgically since otherwise a poor joint function or ulnohumeral instability as described by Givon et al. is likely [21–23]. Our results support this thesis. Patients with an accompanying injury of the coronoid process showed higher pain levels, less satisfaction, worse functional outcome and worse PROM scores.

Comparing the subgroups A and B we observed a significantly decreased extension capability in group A. The further functional parameters did not differ between these groups. Other studies by Korner, Shore and Hamaker et al. describe a worse functional outcome also affecting the flexion capability [14–16]. A potential reason could be shorter FU timepoints with less time for rehabilitation.

The aforementioned long-term effects (improvement in OES and patients’ satisfaction over time) might be due to decreasing pain levels and a better habituation of the patient to the altered functionality of the elbow. Also, patients with an injury that was not affecting the dominant arm usually describe less problems during daily life. While there were no significant differences in the used surgical treatment and approach, various factors such as a heterogenic aftercare, unknown compliance level and comorbidities (such as abuse of alcohol or nicotine, existing preconditions and medication) as well as patient’s age might have had a negative influence on the rehabilitation process.

In order to further address this problem, a study with a much larger patient collective would be necessary. Also, a long-term follow-up after 7–10 years might yield additional results. However, since MF and MLL are rare entities of elbow injuries this seems to be ideal in a multicentre approach.

### Limitations

Limitations of our study include the retrospective study design, the small number of patients (*n* = 35) and the heterogeneity in respect to aftercare, timepoint of follow-up and patient age. However, since MF and MLL are a rare entity of elbow injuries a bigger patient cohort is scarcely available and could only be recruited in a multicentre approach.

## Conclusion

The presented data did not support the preliminary hypothesis. The hypothesis of an injury equivalent to syndesmosis lesions of the ankle joint (besides Essex-Lopresti injuries) cannot be confirmed at present. Instead, our results were congruent to existing findings in literature confirming that fractures with a close relation to the joint tend to have a worse outcome. Especially accompanying fractures of the coronoid process seem to be negative prognostic factors. While in our current study only the extension after rehabilitation displays significant changes, additional correlations might yet not be revealed due to an insufficient number of patients. Further research should therefore focus on larger patient cohorts.

## Data Availability

The datasets used and/or analysed during the current study are available from the corresponding author on reasonable request.

## Abbreviations

DASH: Disabilities of Arm, Shoulder and Hand
LCP: Locking compression plate
MEPS: Mayo Elbow Performance Score
MF: Monteggia fracture
MLL: Monteggia-like lesion
NRS: Numeric rating scale
OES: Oxford Elbow Score
PROM: Patient-reported outcome measurement
PURJ: Proximal ulno-radial joint
ROM: Range of motion
VAS: Visual analogue scale
